# Prevalence of hepatitis B and C in patients with meningiomas and glioblastoma multiforme

**DOI:** 10.3892/ol.2013.1126

**Published:** 2013-01-10

**Authors:** MARC B. CABANNE, QUANG D. MA, LILLIAN MECUM, RAHUL JANDIAL, JAVED SIDDIQI, MIKE Y. CHEN

**Affiliations:** 1 Division of Neurosurgery, Arrowhead Regional Medical Center, Colton, CA 92324;; 2 Division of Neurosurgery, City of Hope National Medical Center, MOB 2001J, Duarte, CA 91010, USA

**Keywords:** glioblastoma, meningioma, hepatitis, epidemiology, prevalence

## Abstract

The prevalence of hepatitis B and C in patients with glioblastoma multiforme or meningiomas has not been described. These infections are known to modulate the activity of the immune system, which potentially influences the development and course of cancer. We hypothesized that chronic hepatitis infection, which activates the immune system, decreases the risk of brain tumors, particularly those that are highly malignant. We performed a retrospective study to examine the prevalence of hepatitis B and C in patients with meningiomas and glioblastomas. The combined prevalence of hepatitis B and C in the USA from 1999–2008 was 5.7%. The prevalence of hepatitis B and C in patients with meningiomas was 2.4%; while among glioblastoma patients, the prevalence of hepatitis B and C was 1.38%. The odds ratio of having hepatitis B or C with glioblastoma versus meningiomas was 0.56, with a confidence interval of 0.19–1.6 and a P-value of 0.29. Compared with historical controls, the prevalence of hepatitis B and C in meningioma and glioblastoma patients was decreased. However, this difference may be attributed to the retrospective nature of our data and the natural history of hepatitis B and C infections. The prevalence of these viral infections was not statistically different in patients with meningiomas and glioblastomas. This suggests that hepatitis B and C primarily influence slow-growing, benign tumors and more aggressive cancers equally, if at all. To definitively test our hypothesis, future studies in which data are prospectively gathered are likely to be required.

## Introduction

Advances in resection techniques and adjuvant therapies continue to improve the outcome of patients with malignant primary brain tumors. However, substantial gains have been slow and the prognosis of patients with glioblastoma multi-forme (GBM), the most common type of primary malignant brain tumor in adults, remains poor. The median survival time following diagnosis is 14 months. A greater understanding of the epidemiology, in particular with regard to immunological factors, may increase insights into more effective therapeutic strategies.

According to the 2000–2004 data of the Central Brain Tumor Registry of the United States (1), GBM accounts for 19% of all primary intraparenchymal brain tumors. Although GBM is relatively rare in younger patients, its incidence increases with age; individuals between 75 and 84 years of age have the highest incidence rates. The median affected age is 64 years and the overall annual incidence rate is 3.1/100,000 individuals. The prognosis remains poor, with a 5-year survival rate of <4% from the time of diagnosis.

The highest incidence of primary brain tumors is due to GBM, second only to meningioma. Similar to with GBM, the incidence of meningioma increases with age. The total number of meningioma cases in the USA between 2004 and 2006 was 53,455, compared with 27,040 cases of GBM. The occurrence of meningioma is 7-fold more common in Caucasians compared with African-Americans, and its frequency of occurrence in females is 2.7-fold higher that in males (1). In contrast to GBM, the overall 5-year survival rate of patients with benign menigiomas is 70%; skull base meningioma patients and younger patients have an even higher survival rate (2).

Given the poor prognosis associated with malignant brain tumors, intense research of the epidemiological and molecular profile of this disease is underway. Established risk factors for the development of primary brain tumors include exposure to ionizing radiation, increasing age, male gender, Caucasian ethnicity and familial tumor syndromes.

The hepatitis B (HBV) and C (HCV) viruses are well characterized. A correlation between hepatitis infection and neoplasia has also been identified, and is reflected in the increased risk of hepatocellular carcinoma observed in patients with HBV/HCV (3–6). It is estimated that ∼4% of individuals in the USA are affected by HBV. The annual incidence represents 200–300,000 new cases in the USA, or an incidence rate of 1/1,359 (0.07%). By comparison, 2–5 million people in the USA are proposed to be affected by HCV, often as asymptomatic carriers. The annual incidence is 150,000 new cases, with a rate of 1/1,813 (0.06%) (7).

A correlation between brain tumors and hepatitis infection is yet to be reported. An impact of co-morbidities, including the effect of HBV/HCV infection on the occurrence and/or progression of GBM, has not yet been described. One line of evidence supporting the hypothesis that a chronic hepatitis infection may decrease the risk of tumorigenesis is epidemiological data suggesting a correlation between immune system status and the risk of gliomagenesis. Population-based studies have focused on the presence of allergic and inflammatory states in patients diagnosed with glioma. Certain studies indicate that the risk of developing glioma is decreased by heightened immune states. Schoemaker *et al* identified a negative correlation between the risk of developing glioma and a history of asthma and hay fever (8). Likewise, Wiemels *et al* demonstrated that patients with allergies were 50% less likely to develop gliomas (9). Additionally, certain infections have been demonstrated to induce a similar effect. For example, patients with gliomas are less likely to have had a herpes virus infection (10). Furthermore, it is also possible that hepatitis infection, by way of immunomodulation, decreases the risk of glioblastoma tumorigenesis independently of the shortened lifespan associated with the viral illness.

The mechanisms underlying these epidemiological observations remain unclear. However, animal studies have clearly indicated that natural killer cell activity, macrophage activation and interferon production following viral infection are increased in the setting of chronic irritation of the immune system, as observed in murine hepatitis infections (11–14). Notably, mice previously infected with murine hepatitis virus demonstrated augmented responses to antitumor chemotherapy, resulting in longer survival times (15). Human studies have also revealed that interferon levels are significantly higher in patients with HCV compared with healthy controls (16). It is well-known that macrophage activity and endogenous interferon levels are key elements in the cancer immunosurveillance response (17). Thus, it is reasonable to expect that a heightened immune state is able to alter glioma formation and/or progression.

Given the epidemiological findings relevant to other viruses and the molecular basis of the immune response, we hypothesize that patients with GBM have a lower incidence of prior hepatitis B/C virus infection compared with the overall population. Conversely, this finding may be interpreted as evidence that patients with a history of HBV/HCV are less likely to be diagnosed with GBM.

## Materials and methods

### Patients

Patient data were obtained by performing a retrospective chart review at two different institutions in Southern California, following IRB approval. The first institution was the City of Hope National Medical Center (COH), a comprehensive cancer center located in Duarte (CA, USA), and the second was the Arrowhead Regional Medical Center (ARMC) in Colton (CA, USA). At both institutions, a search of the electronic medical record was performed for all patients diagnosed with GBM. Data were available from 1998–2009 at the COH and 2001–2010 at the ARMC. Only patients with a tissue diagnosis of GBM were considered in the study. When a patient was identified as having GBM, the remainder of the patient’s medical record was explored, including past laboratory studies, serological testing and the patient’s response to medical history questionnaires included within the medical record. As our comparison group, HBV/HCV infection was also measured in patients with meningiomas, in the same manner that patients with GBM were evaluated. Given the mostly benign pathology of meningiomas, patients may elect not to undergo biopsy or resection. Thus, patients with a radiographic diagnosis of meningioma in addition to tissue diagnosis were included in the study.

### Statistical analysis

Given the study design, which included cases and controls, we deemed an odds ratio to be an appropriate measure of relative risk. The incidence of meningiomas with or without a history of HBV/HCV served as a control in order to compare and calculate an odds ratio, and to estimate the relative risk of GBM with a prior hepatitis infection. The odds ratio was calculated by dividing the number of positive HBV/HCV patients in the GBM population (GP) by the number of negative HBV/HCV patients (GN); this number was then divided by the number of positive HBV/HCV patients in the meningioma population (MP) divided by the number of negative HBV/HCV patients (MN). Thus, odds ratio = (GP/GN)/(MP/MN). An odds ratio of 1 implies there is an equal probability of HBV/HCV in GBM and meningioma. An odds ratio of >1 suggests a higher probability of having HBV/HCV and GBM compared with meningioma. Conversely, an odds ratio of <1 suggests a lower probability of having HBV/HCV and GBM versus meningioma. P<0.05 was considered to indicate a statistically significant result.

## Results

**Figure 1:**
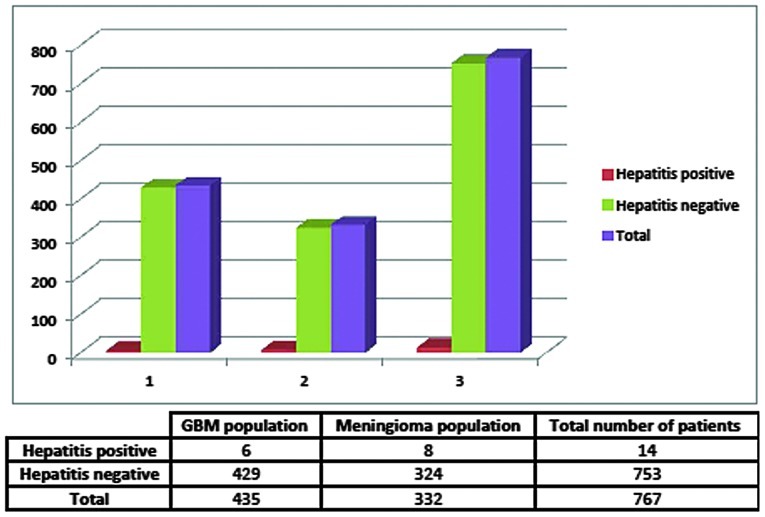
Patient data. The number of patients in each population of the study, and whether or not they were found to have a history of, or an active, hepatitis infection. The GBM population was larger than the control meningioma population, however fewer patients in the GBM population were found to have a hepatitis infection. GBM, glioblastoma multiforme.

**Figure 2:**
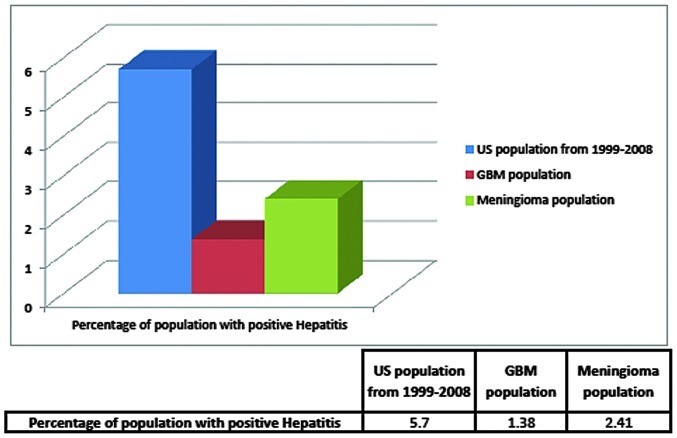
Percentage prevalence of hepatitis infection. The prevalence of hepatitis infection in both patient populations in this study compared with the prevalence of hepatitis infection in the US population (22). The GBM population demonstrates the lowest rate of hepatitis infection. Both brain tumor populations have substantially lower hepatitis infection rates when compared with the US population during the same time period. GBM, glioblastoma multiforme.

Between 2001 and 2010, 76 ARMC patients were identified as having GBM, 3 of whom also had positive anti-HCV antibody serologies and no positive HBV serologies. At the COH, between 1998 and 2010, 359 patients had GBM and again only 3 were found to have HBV or HCV. Retrospectively, the overall prevalence of any type of hepatitis virus within this combined GBM population was 1.38%, or a rate of 0.0138. As a control, the rates of hepatitis infection in patients with meningioma were also examined at both institutions. Of the 332 meningioma patients, 8 had either active infection or a history of hepatitis infection, producing a rate of 0.0241, or 2.4%, of the meningioma population (Figs. 1 and 2). The odds ratio of having HBV or HCV with GBM compared with HBV or HCV with meningiomas was 0.566 with a 95% confidence interval of 0.194–1.648 (Table I). A χ^2^ test of association revealed a Pearson’s value of 1.115 (P=0.29) and a one-tailed Fisher’s exact probability of P=0.21.

## Discussion

To the best of our knowledge, this is the first investigation of the correlation between GBM and hepatitis virus infection. The main limitations of our study are two-fold. The first limitation is that this was a retrospective analysis. Therefore, patients in this study were not prospectively tested or specifically asked about hepatitis status at the time of diagnosis of GBM or meningioma, and thus the incidence of infection may be underreported. The second limitation was the small sample size relative to the number of patients who were expected to have GBM and hepatitis B/C if the two diseases did not preclude each other. The discordance between the co-diagnosis of GBM and hepatitis is that patients with hepatitis often have associated medical or social factors that predispose them to shorter life spans and thus are less likely to be diagnosed with GBM, a condition associated with increasing age. This may further explain the lower-than-expected prevalence of HBV/ HCV in our study populations.

Our study limitations allows us to only calculate an odds ratio, which is not necessarily a relative risk, which is the most accurate value for correlational statistics. However, given our sample size, our limitation is that we are assuming odds ratio is approaching a relative risk if it was able to be determined. In other words, we are assuming the two values are similar for this study. Our original hypothesis was that a prior hepatitis B or C infection would confer resistance to GBM, and thus patients with hepatitis B and C would have a lower relative risk of contracting the disease. However, calculation of the relative risk is flawed by the retrospective nature of the study in which we evaluated GBM populations rather than undertaking a prospective analysis of hepatitis B and C populations. Also, it is noteworthy that relative risk in this instance is only applicable if hepatitis infection occurred prior to the diagnosis of GBM or meningioma. Although not all patient information relevant to the timing of hepatitis infection was available, it is almost certain that hepatitis infection occurred prior to GBM, due to the rapid progression and lethality of the disease. However, this assumption may be invalid for meningioma patients whose disease typically occurs over a chronic course. Nonetheless, although our findings are not statistically significant, we propose that further, statistically powerful prospective studies are required to investigate the correlation between hepatitis infection status and GBM.

It may be speculated that currently unidentified factors underlie the lower incidence of HBV/HCV observed in GBM patients and that infection with one of these two viruses provides a form of immunoprotection against the development of GBM. One possible mechanism to support this idea is that patients with a chronic hepatitis infection demonstrated elevated levels of interleukin 13 (IL13). IL13, a potent mediator of apoptosis in tumor cells, has been demonstrated to be significantly increased in patients with chronic hepatitis infection and allergies (18,19). High grade gliomas frequently overexpress an IL13 receptor subtype, designated IL13Rα2. IL13Rα2 functions as a ‘decoy receptor’ and is ineffective in activating the apoptotic cascade upon ligand binding. We speculate that hepatitis infections may increase IL13 levels, dampening the competitive effects of IL13Rα2 and thereby inhibiting gliomagenesis.

Glioma progression is also associated with immunosuppression in both murine models and patients. This state of immunosuppression is characterized by a diminished capacity of macrophages and lymphocytes to secrete interleukins and tumor necrosis factor α (TNFα) (20,21). Therefore, increased circulating levels of TNFα released as a consequence of hepatitis-induced stimulation of the immune system may diminish tumor viability.

In summary, there is limited epidemiological evidence in support of a role for HBV/HCV in GBM tumorigenesis and/or progression. In our study, we observed a lower incidence of HBV/HCV in GBM patients compared with the general population, suggesting that HCV/HBV may interfere with GBM development. However, there was no statistically significant difference when compared with the incidence of hepatitis in meningioma patients that served as a control group.

Identifying factors that are able to influence gliomagenesis may substantially increase our understanding of GBM biology and enhance the treatment of patients affected by this invariably life-threatening disease. We suggest that a future prospective study following patients with HCV and HBV to assess their rates of acquired GBM compared with non-hepatitis patients, in addition to prospectively screening patients with GBM for HCV and HBV serological markers, is required.

## Figures and Tables

**Table I:** Statistical analysis.

Condition and population	Rate (%)	Test	Result	95% confidence interval
Hepatitis in GBM population	1.38	Odds ratio	0.566	0.195–1.649
Hepatitis in meningioma population (control)	2.41	Fisher’s exact probability	P=0.21	

In addition to showing the rates or prevalence of hepatitis infection in the two brain tumor populations, Table I demonstrates the risk ratio and odds ratio for having GBM with a previous hepatitis infection, using the meningioma population as a control. The odds ratio is used to estimate relative risk. With an odds ratio of 0.566, this suggests there may be a lower probability of having a previous hepatitis infection with GBM than having a previous hepatitis infection with meningioma, although this difference was not statistically significant. GBM, glioblastoma multiforme.
